# Loss of reef roughness increases residence time on an idealized coral reef

**DOI:** 10.1038/s41598-022-24045-4

**Published:** 2022-11-12

**Authors:** M. Lindhart

**Affiliations:** Department of Civil and Environmental Engineering, Stanford University, 473 Via Ortega, Stanford, CA 94305 USA

**Keywords:** Marine biology, Physical oceanography

## Abstract

Through idealized, numerical models this paper investigates flows on a reef geometry which has received significant attention in the literature; a shallow, fringing reef with deeper, shore-ward pools or lagoons. Given identical model geometries and varying only reef flat drag coefficients between model runs ($C_D = [0.001,0.005,0.01,0.05,0.1]$), two distinct circulation patterns emerge. One is related to low reef water levels and high roughness, and efficiently flushes the entire reef system resulting in low residence times (an ‘open reef’). The other is related to high reef water levels and low roughness, and in spite of the development of an offshore undertow, this dynamic is inefficient at flushing the reef-pool system and facilitating exchange flow with offshore waters (a ‘closed reef’). This paper shows that even given indistinguishable geometry and offshore conditions, this information is insufficient to predict reef dynamics, and suggests that reef roughness (and thus reef health) plays a comparable role in determining circulation patterns and residence times. Furthermore, a transition from open to closed or vice versa caused by e.g., a loss of reef roughness or increase in mean sea level could have implications for transport and mixing of nutrients and water masses, as well as larval dispersal.

## Introduction

The present study investigates the effect of varying reef roughness on dynamics and residence times on an idealized reef. Wave-driven flows on coral reefs are typically described by a one-dimensional (1D), depth-averaged model, which details the approach and breaking of waves on a shallow fore-reef or reef crest, and estimates the onshore or cross-reef flow (see e.g.^1,2^). Reference^3^ suggest that the two end members of this 1D balance are the ‘open reef’, described by balancing an onshore pressure gradient force by bed friction, and the ‘closed reef’, in which an onshore radiation stress gradient is balanced by an offshore pressure gradient force. The 1D model gives a satisfactory description of some observed flows (e.g.^1,4,5^), however, the horizontal velocity profile is unaccounted for, and some observations reveal vertically sheared flows similar to beach undertow that cannot be captured by the existing model^6–9^. While a deviation from the established 1D model is both expected and documented, so far no framework has been presented to interpret this departure and its implications for reef-wide circulation.

Undertow has been observed and described as it manifests on sandy beaches (e.g.^10,11^) where the cross-shore mass flux is zero. However, reef systems often exhibit alongshore bathymetric variability allowing for a nonzero flux, the effect of which was studied by Ref.^12^, who showed through numerical simulations that an increased onshore mass flux was correlated with a decrease in undertow. Reference^6^ observed in experiments and simulations that undertow decreased as roughness increased, and Refs.^8,9^ observed that reef undertow coincided with high water levels, and ceased during low tide.

With Ref.^3^ as a theoretical framework, this paper shows that undertow may develop on closed reefs (where cross-shore pressure and radiation stress gradients balance and cross-shore mass flux is reduced), and that the associated circulation results in markedly longer residence times. “Materials and methods” section describes the theoretical foundation and the numerical model used in this study, and the “Results” section presents the model results and establishes the connection between the reef flat momentum balance, the horizontal velocity profile (and undertow), and reef-wide circulation patterns and residence times. Finally, the “Discussion” section discusses the implication of the open and closed reef dynamics and the effect of reef degradation on circulation.

## Materials and methods

### Theory of reef dynamics

The 1D depth-integrated, wave-averaged momentum equation on a shallow reef flat in the cross-reef (*x*) direction is given by^13^

$$\begin{aligned} \frac{\partial hu}{\partial t}+\frac{\partial hu^2}{\partial x}=-gh\frac{\partial {\overline{\eta }}}{\partial x}-\frac{1}{\rho }\frac{\partial S_{xx}}{\partial x}-\frac{\tau _b}{\rho }, \end{aligned}$$

where *h* is the depth, ${\overline{\eta }}$ is the wave setup, *u* is the depth-averaged, Lagrangian velocity, $\rho$ is the density, $S_{xx}$ is the radiation stress (assuming normal incident waves), and $\tau _b$ the bed shear stress (assuming no wind stress). For a tidally modulated, wave-averaged flow, local accelerations are small, and in the absence of large spatial gradients, advection is negligible. Thus, three momentum terms remain,

1 $$\begin{aligned} -gh\frac{\partial {\overline{\eta }}}{\partial x}-\frac{1}{\rho }\frac{\partial S_{xx}}{\partial x}-\frac{\tau _b}{\rho }=0. \end{aligned}$$

From left to right, these are the pressure gradient force (PGF), the radiation stress gradient (RSG), and the bottom friction (BF) terms. In Ref.^3^, we considered two scenarios in which the cross-shore PGF is balanced by either the BF or the RSG, and classified these as either ‘open’ or ‘closed’. That is, open reefs are governed by an onshore pressure gradient force which drives flow across the reef and is balanced by bottom friction, i.e. $\Vert \frac{gh\frac{\partial {\overline{\eta }}}{\partial x}}{\frac{1}{\rho }\tau _b}\Vert \approx 1$. A closed reef is governed by an onshore radiation stress gradient, and balanced by an offshore pressure gradient, $\Vert \frac{gh\frac{\partial {\overline{\eta }}}{\partial x}}{\frac{1}{\rho }\frac{\partial S_{xx}}{\partial x}}\Vert \approx 1$.

#### Open reef vertical structure of flow

Given a balance between the PGF and BF, the velocity profile is^14^

2 $$\begin{aligned} u(z)=\frac{u_*}{\kappa }\ln \left( \frac{z-d}{z_0}\right) , \end{aligned}$$

where $u_*$ is the friction velocity, *z* is the vertical coordinate, $z_0$ the roughness height, the von Karman constant is $\kappa =0.41$ (following^15^), and the displacement height *d*, which we will neglect for simplicity. Thus, in the absence of a significant RSG in Eq. (1), the flow is described by a logarithmic, unidirectional velocity profile. For example, Ref.^16^ found the logarithmic profile to fit observed horizontal velocity profiles well on locations of a reef flat downstream of the surf zone where the PGF balanced the BF.

#### Closed reef vertical structure of flow

In contrast to the logarithmic velocity profile, there is no widely acknowledged analytical solution to Eq. (1) in the case that the RSG is non-negligible. One suggested solution was developed by Ref.^10^ and evaluated against field observations by Ref.^9^. Here,

3 $$\begin{aligned} u(z)=A\ln \left( \frac{h-z}{h-z_0}\right) +B\ln \left( \frac{z}{z_0}\right) +U_0, \end{aligned}$$

where

$$\begin{aligned} A= & {} \frac{h}{\rho \nu _0}\frac{\partial F }{\partial x},\\ F= & {} \frac{g\overline{\eta '^2}}{2} + \frac{R}{\rho },\\ B= & {} \frac{\tau _b h}{\rho \nu _0}. \end{aligned}$$

**Figure 1:**
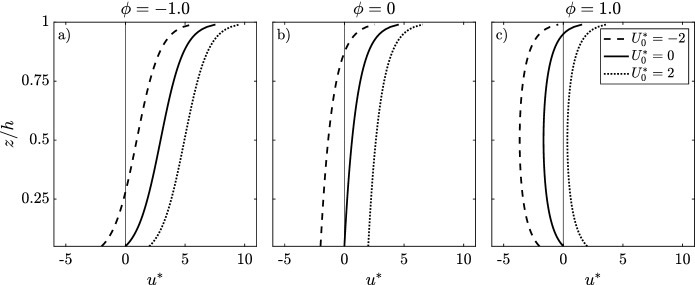
Velocity profiles following Refs.^9,10^, where $z_0=0.05h$. $\phi$ is evaluated in the range $\phi \in \left[ - 1,0,1\right] .$

$\overline{\eta '^2}$ is the mean square of the surface elevation, *R* the contribution due to rollers, and $U_0$ can be found by matching to observations. Thus, *F* is the radiation/forcing imbalance^9^. In this derivation, $\nu _0$ is the representative strength of a parabolic vertical eddy viscosity. For details on how to estimate each term in Eq. (3), see^9^. Here, the specifics of calculating the terms are not of concern, but rather the interpretation of Eq. (3) as the resulting velocity profile of a balance between the PGF, BF, and RSG terms, where bottom friction and wave forcing introduce a shear stress at the bottom and surface boundaries, respectively. Equation (3) can be nondimensionalized by A such that

4 $$\begin{aligned} u^*=\ln \left( \frac{h-z_0}{h-z}\right) +\phi \ln \left( \frac{z}{z_0}\right) +U_0^*, \end{aligned}$$

where $u^*=\frac{u(z)}{A}$, $U_0^*=\frac{U_0}{A}$, and $\phi =\frac{B}{A}=\tau _b\left[ \frac{\partial F}{\partial x}\right] ^{-1}$ is a dimensionless parameter scaling the relative contribution of bottom friction to forcing from waves and surface slope. In Fig. 1, solutions to Eq. (4) are given for different values of $\phi$ and $U_0^*$.

Here, the effect of a surface and bottom shear force on the velocity profile are shown. The magnitude and vertical extent of the undertow depends on the ratio $\phi$, related to the ratio of momentum terms, and $U_0^*$, related to the mass flux. For example, while $\phi =0$ (negligible bottom friction $\tau _b=0$, Fig. 1b) implies a ’beach-like’ dynamic or a closed reef, the development of an offshore undertow is not conclusive, but depends on $U_0^*$. Reference^10^ offers no clear method to evaluate $U_0$, hence, the theory detailed above is not predictive as to the establishment and magnitude of an undertow, and future work on this subject seems warranted, in particular on rough reef environments.

### Numerical model

**Figure 2:**
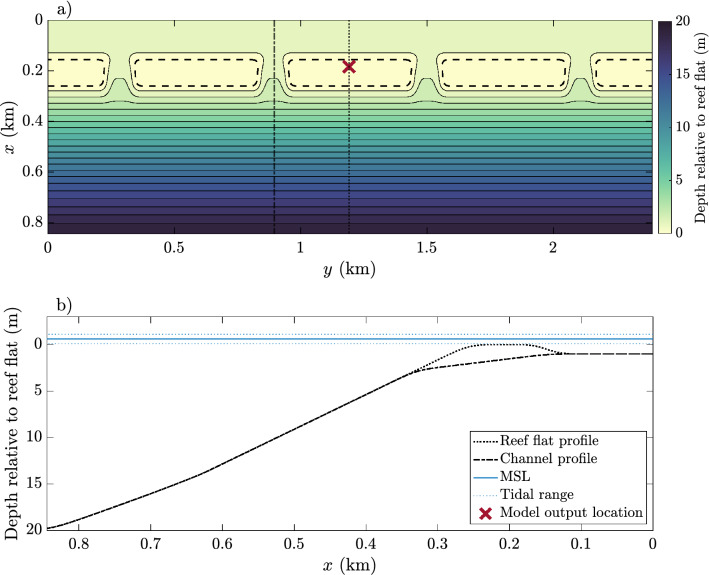
Model domain. (**a**) Bathymery. The area within the bold, dashed lines indicates the reef flats and the part of the domain where the drag coefficient is varied between model runs. Vertical dotted (dot-dash) line indicates profile along the reef flat (channel) in (**b**). The red marker shows the location of model outputs in Figs. 8 and 9. (**b**) Depth profile of the model domain, mean sea level (MSL) and tidal range.

The numerical model is a coupled Delft3D FLOW and SWAN wave model^17,18^ using structured grids. The bathymetry is shown in Fig. 2. The main feature of the model is a series of shallow reef flats (mean depth 0.6 m) with equidistant, cross-reef channels, creating an idealized model of a reef similar to those studied in Ofu, American Samoa, by Refs.^3,19^, as well as the numerical models by Refs.^20,21^. The model spans approximately 0.85 km by 2.4 km, with a horizontal grid size of 8 m, and has 10 equally spaced, vertical $\sigma$-layers (each occupying $10\%$ of the water column).

The roughness is prescribed by a drag coefficient, $C_D$, where $C_D=0.01$ everywhere but the horizontal reef flat, shown in Fig. 2a as the area enclosed by the dashed line. The drag coefficient on the horizontal reef flat is varied between model runs where $C_D=[0.001, 0.005, 0.01, 0.05, 0.1]$, where a low value of $C_D$ indicates low coral cover, and a higher value indicates high coral cover. Thus, in the third of these cases the drag coefficient is $C_D=0.01$ everywhere. Note, however, that factor other than coral cover can contribute to increased roughness. Reference^22^ found that a spatially uniform roughness parameter was sufficient to model flow in Kaneohe Bay, Hawaii, in spite of clear spatial variability in coral cover between the reef flat and lagoon. They suggested that bottom features such as patch reefs, outcrops, and craters and mounds from shrimp and worms could contribute to high friction in lagoons and channels. As such, it may be difficult to delineate between reef flats and lagoons and high and low roughness regions on a physical reef. In this idealized numerical study these additional contributions to roughness are neglected, and roughness is exclusively considered a proxy for coral cover.

Thus, the drag coefficients in this study are chosen to span a wide range of reefs. $C_D=0.001$ and $C_D=0.1$ represent the smallest and largest drag coefficients observed on reefs, respectively. Estimated drag coefficients have been found to span orders of magnitude on real coral reefs^23,24^, e.g., $C_D\approx 0.006{-}0.08$ in Moorea, French Polynesia^2^, $C_D\approx 0.05$ in Chagos^25^, and $C_D\approx 0.03{-}0.06$^26^ and $C_D\approx 0.01{-}0.1$^19^ on different parts of the same reef system in Ofu, American Samoa. The results found by Refs.^2,19^ are interesting as estimates of $C_D$ vary by an order of magnitude not only on the same reefs, but over a distance of 100 m or less. To the author’s best knowledge, there is no widely accepted method of predicting drag coefficients a priori, although^19^ proposed a geometry-based approach to evaluate $C_D$ that correlated well with hydrodynamically derived drag coefficients in both the original paper and in Ref.^25^. Adding to the complexity, Ref.^24^ showed that $C_D$ varies with depth, and therefore varies in time due to tides. While this may be important in particular on shallow reefs, the effect of depth on hydrodynamic roughness is not considered in this paper.

In 3D, the bed shear stress is defined

$$\begin{aligned} \vec {\tau }_b=\rho _0 C_D \Vert \vec {u}_b \Vert \vec {u}_b, \end{aligned}$$

where $\vec {u}_b$ is the velocity in the bottom layer^27^. A $k-\epsilon$ turbulence model is used to calculate vertical eddy viscosity, and wave enhanced bed shear stress is estimated according to Ref.^28^. Energy lost to breaking is converted to roller energy and dissipated over shallow regions, see^27^.

All models are run over a tidal cycle of 12 h, where the mean sea level (MSL) relative to the reef flat is 0.6 m and the tidal amplitude is 0.5 m, defined at the offshore boundary. All other boundaries are closed. Additionally, the offshore boundary is forced by a JONSWAP spectrum with $H_s=1$ m, $T=10$ s, and a peak enhancement factor of 3.3. Wave breaking is calculated according to Ref.^29^, with a breaking parameter of $\gamma =0.75$. The FLOW model has a time step of three seconds, and the communication time step between the FLOW and WAVE models is 4 min. A one hour smoothing period provides stability while adjusting to initial and boundary conditions, and the model is run for an additional 5 h to stabilize before saving model outputs, i.e., the first 6 h of the model were discarded.

The geometry and forcing of the numerical model roughly represents the southern reefs of Ofu, American Samoa, which have been studied in detail by Refs.^3,8,9,19,26,30,31^. Additionally, the fringing reef is a commonly occurring geometry, see e.g., Ningaloo Reef^5,32,33^, Oahu^34^, Guam^35^, Faga’alu Bay^36^, and many others.

### Particle tracking

Particle tracking is calculated in post processing using the simulated 3D velocity fields and vertical eddy viscosity of the $k{-}\epsilon$ turbulence model. A horizontal eddy viscosity of $K_h=0.5$ m$^2/$s is assumed. While the quantitative results in this study depend somewhat on the choice of $K_h$, the qualitative dynamics and conclusions do not, and as it is not the intention to reproduce observations, the specific choice of $K_h$ is not considered further here. The value of $K_h$ is of similar order of magnitude as Refs.^37,38^ found on coral reefs. Following e.g.^39^, the velocity field is seeded with non-inertial tracer particles at a time $t_0$ and their location in space, $\vec {r}(t)$, is propagated using a 3D advection–diffusion model,

5 $$\begin{aligned} \vec {r}(t+\Delta t)=\vec {r}(t)+\Delta t\left[ \vec {u}+\sqrt{\frac{6K_h}{\Delta t}}\vec {d}_h + \left( w+\sqrt{\frac{6K_v}{\Delta t}}d_v+\frac{\partial K_v}{\partial z}\right) \vec {e}_z\right] . \end{aligned}$$

Horizontally, the position at time step $t + \Delta t$ depends on advection by the horizontal velocity, $\vec {u}$, and diffusion where $K_h$ is the (constant) horizontal eddy viscosity, and $\vec {d}_h$ is a vector of random numbers uniformly distributed between $-1$ and 1. In the vertical, the particle is advected by $\Delta t w\vec {e}_z$ where *w* is the vertical velocity and $\vec {e}_z$ the vertical unit vector. Vertical diffusion is determined by the modeled eddy viscosity, $K_v$, and $d_v$, which is a random number uniformly distributed between $-1$ and 1. Two particles are initiated per cell (40 cross-shore cells, 101 along-shore cells, and 10 vertical cells: 80,800 particles in total) and the time step is $\Delta t=24$ s. This calculation is carried out twice (initiated an hour before high tide and an hour before low tide) to capture tidally dependent circulation patterns.

### Residence times

Reef geometry varies significantly between sites, and thus residence times must be calculated with care. For example, Ref.^39^ studied a numerical model of the enclosed Mururoa lagoon, French Polynesia, in which an atoll and distinct passages defined the lagoon relative to the surrounding ocean. This geometry lends itself well to bulk estimates such as turnover time $T=V/Q$^40^ where *V* is the lagoon volume and *Q* the net flow defined at the passages. In comparison, Ref.^36^ studied Faga’alu Bay, American Samoa, where the demarcation between the reef inflow and outflow was less pronounced. Using Lagrangian drifters, they defined residence time by the velocity of a drifter and the distance traveled, normalized by a representative area over which the reef had similar characteristics, thereby estimating spatial residence times of different patches of the reef.

The reef in this study is a chain of shallow reefs with shoreward pools (Fig. 2), and while dominant circulation patterns are present, delineating individual reef systems or circulation cells is ambiguous. Thus, a bulk estimate such as the turnover time is not particularly useful here, since there is no clear area over which to estimate the volume and flow rate. Instead, residence time at a point is defined as the time it takes for a water parcel initiated in that point to leave the reef, following the definition in Ref.^40^. The reef domain is seeded with particles, and particle trajectories are calculated with the advection–diffusion Eq. (5). When the particles have moved just offshore of the reef, the time elapsed is noted and attributed to the original starting point. Residence times are averaged over depth, giving a 2D horizontal spatially varying estimate.

## Results

In this section, results of the numerical models are presented. Please refer to the Supplementary Materials, which show the trajectories of 20 particles released in the low friction model (Video 1) and high friction model (Video 2) on high tide. Video 1 is an example of a closed reef, and Video 2 an example of an open reef.

### Reef circulation and vertical structure of flow

**Figure 3:**
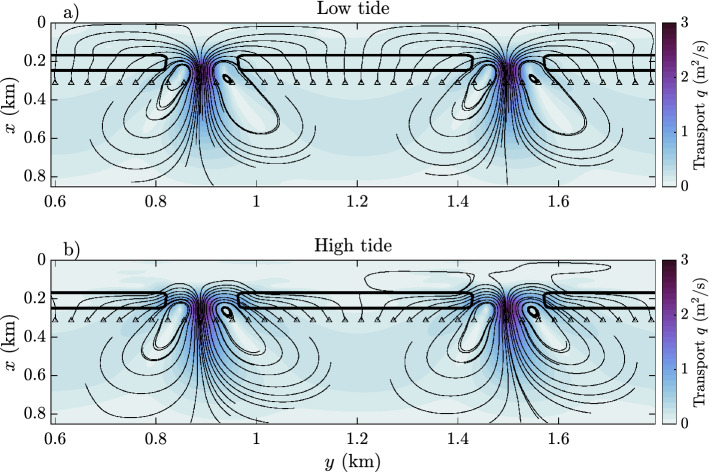
Streamlines of Lagrangian transport on (**a**) low tide and (**b**) high tide for the $C_D=0.001$ model.

The widely studied reef model in e.g., Refs.^22,41^, describes setup from wave breaking, creating a cross-reef pressure gradient that drives a flow onshore on the shallow reef flats. This flow is directed into shallow pools and lagoons, and led out deeper, intercepting channels into the ocean. On low tide, across all models, this is the dominant circulation pattern. An example of this is found in Fig. 3a, showing the model results from the $C_D=0.001$ model on low tide (water level equivalent to a 0.1 m depth on the reef flat in the absence of waves). Note in particular that streamlines are directed perpendicular (or cross-reef) on the reef flats, and pass through the pools. On high tide, the same model shows a significantly different circulation pattern (Fig. 3b, water level equivalent to a 1.1 m depth on the reef flat in the absence of waves). Here, the streamlines are directed almost parallel to the reef flats (alongshore), exiting directly into or near the channels and bypassing the pools almost entirely. The streamlines that do enter the pools show entrapment in local vortices and do not exit the channels. The colored contours show that the Lagrangian depth-averaged transport is generally larger on the reef flat and in the pools on low tide.

**Figure 4:**
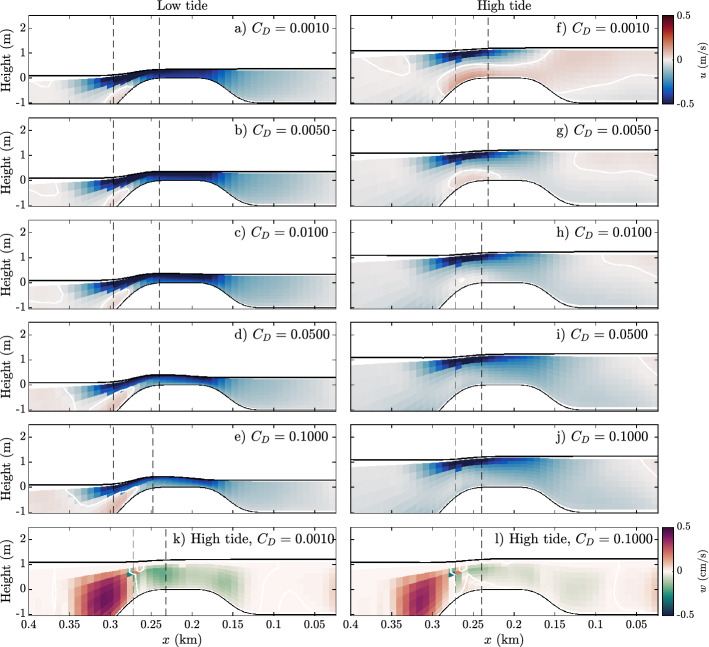
(**a–j**) Cross-reef Lagrangian velocity. Red indicates flow moving to the left, blue indicates flow moving to the right. The black, solid lines show the water level and bathymetry. (**k–l**) Vertical velocities on high tide for the models with the highest and lowest drag coefficients. Red colors indicate upward velocities and green colors indicate downward velocities. In all figures the vertical, dashed lines show where the majority of wave breaking takes place.

Across the reef (reef flat profile in Fig. 2), the Lagrangian velocity for all five models on both low and high tide are shown in Fig. 4. On low tide, the model results are similar; wave breaking creates a strong cross-reef, unidirectional flow. The horizontal circulation patters on low tide for all models are similar to Fig. 3a. Note that the cross-reef surface slope increases with increasing friction; as the opposing friction increases, so does the pressure gradient force. On high tide, two different patterns emerge; one which is similar to the low tide dynamics (Fig. 4h–j) and one that shows a significant offshore return flow, or undertow, on the reef flat (Fig. 4f,g). Hence, as the depth increases or the roughness decreases, the offshore undertow increases, as was observed on similar reefs in Refs.^6,9^.

### Particle tracking

**Figure 5:**
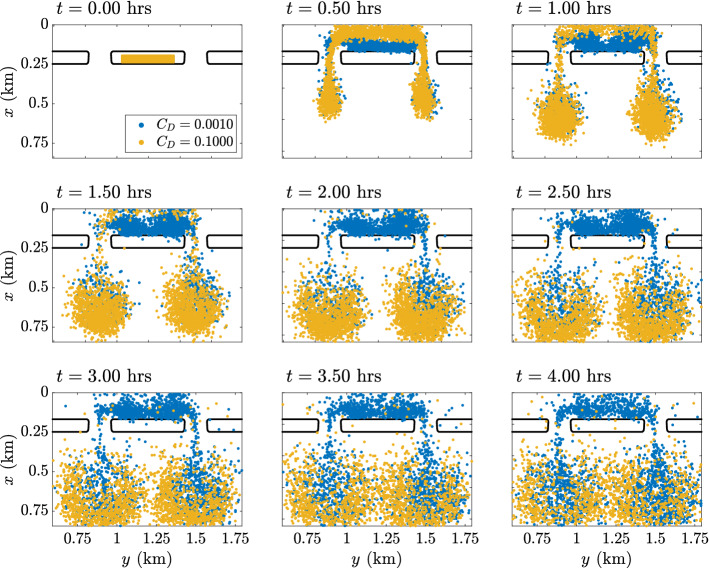
Particle locations at half hour intervals, initiated an hour before high tide. Blue markers: $C_D=0.001$ reef flat roughness, yellow markers: $C_D=0.1$ reef flat roughness. Here, $K_h=0.5\, \text {m}^2/\text {s}$, see Supplementary Materials Figs. 1 and 2 for the same calculation carried out with $K_h=0.1\, \text {m}^2/\text {s}$ and $K_h=1\, \text {m}^2/\text {s}$.

Seeding the simulated velocity fields with particles and propagating them in time and space using Eq. (5), the particle locations are shown at half hour intervals in Fig. 5. Results shown here are for the two models with the smallest and largest reef flat drag coefficients ($C_D=0.001$ and $C_D=0.1$). The particles are initiated an hour before high tide on the reef flat and tracked for four hours.

Compare the particle locations to the Lagrangian velocity field in Fig. 3a,b. In the case of high reef roughness (yellow markers), the particles are transported into the pool and out the channels as the cross-reef flow is directed entirely onshore. After about 2 to 3 h, the majority of particles have exited through the channels and are carried offshore. Some particles in the low roughness model (blue markers) are carried offshore immediately after release $t=0.5$ due to the strong undertow (Fig. 4f). However, strong vertical mixing just offshore of the reef flat (Fig. 4k) transports particles up in the water column and recirculates them onto the reef flat and into the pools. Thus, the majority of these particles do not escape the reef immediately, in spite of the undertow. Once in the pool behind the reef flats, particles are captured in the two horizontal circulation cells identified in the lower figure in Fig. 3. The particles captured here are retained until low tide, where circulation is similar to the high roughness models (Fig. 3a). Thus, in spite of a strong offshore undertow on the reef flat itself in low roughness models, this dynamic is inefficient at flushing the reef-pool system and facilitating exchange flow with offshore waters.

### Residence times

**Figure 6:**
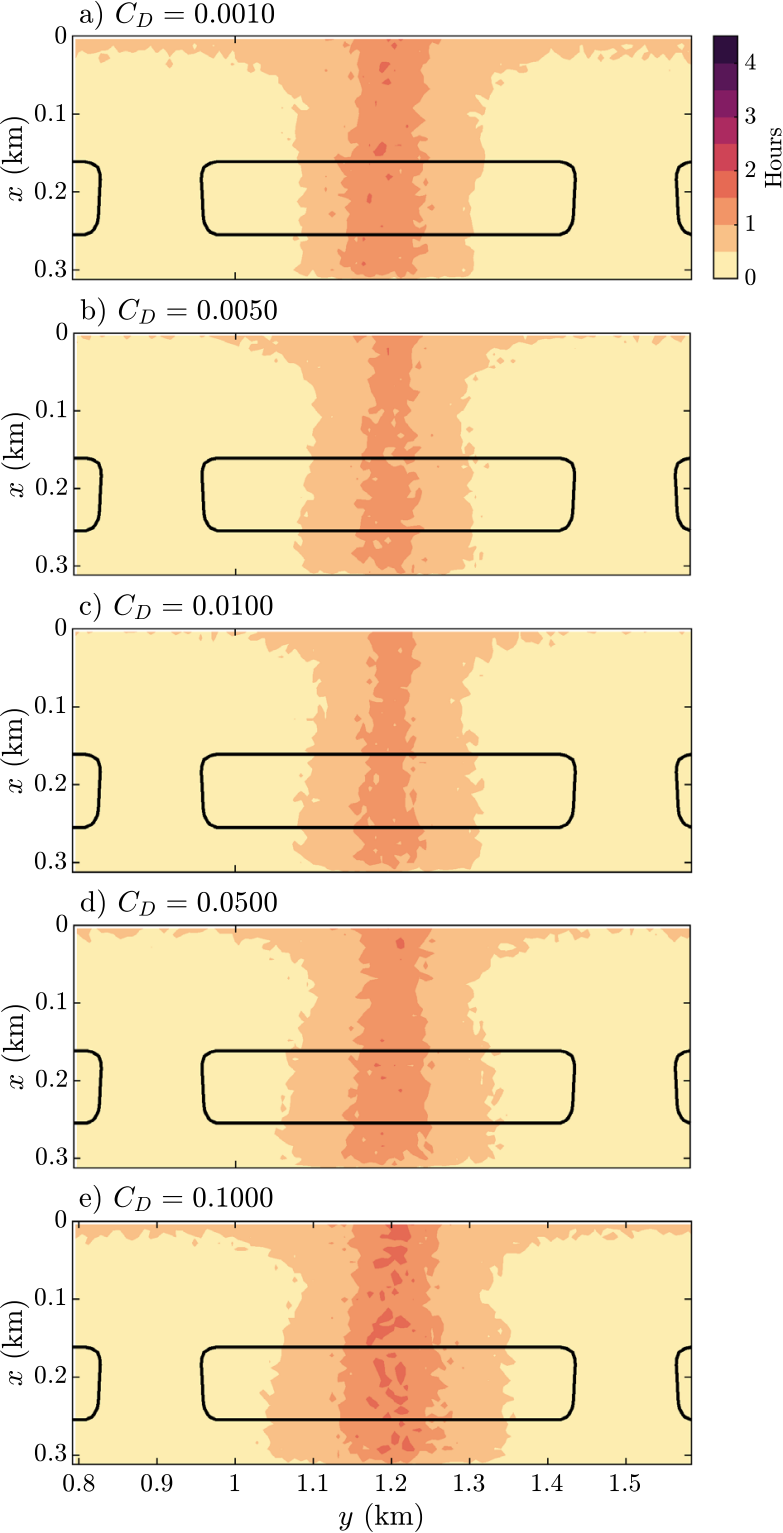
Depth-averaged residence times of particles released an hour before low tide. Solid black lines indicate reef flat location.

Here, particles are initiated in all points across the reef flat, pools, and channels, as apposed to the reef flat only. Particles are tracked, and the time at which a particle leaves the system is noted (defined as their position along the *x*-axis exceeding 0.3 km). Particles are released an hour before low tide and tracked for four hours, and the residence times are found in Fig. 6. The residence time across all models is similar and is largely unaffected by the change in roughness, and most particles have left the system after 1 to 2 h.

Compared to Fig. 4a–e, the similarity in residence times is unsurprising as the circulation patterns across all models are similar. Particles initially located close to the channels are immediately transported offshore, and residence times increases with distance from the channels. Residence times are almost entirely determined by the along-shore position.

**Figure 7:**
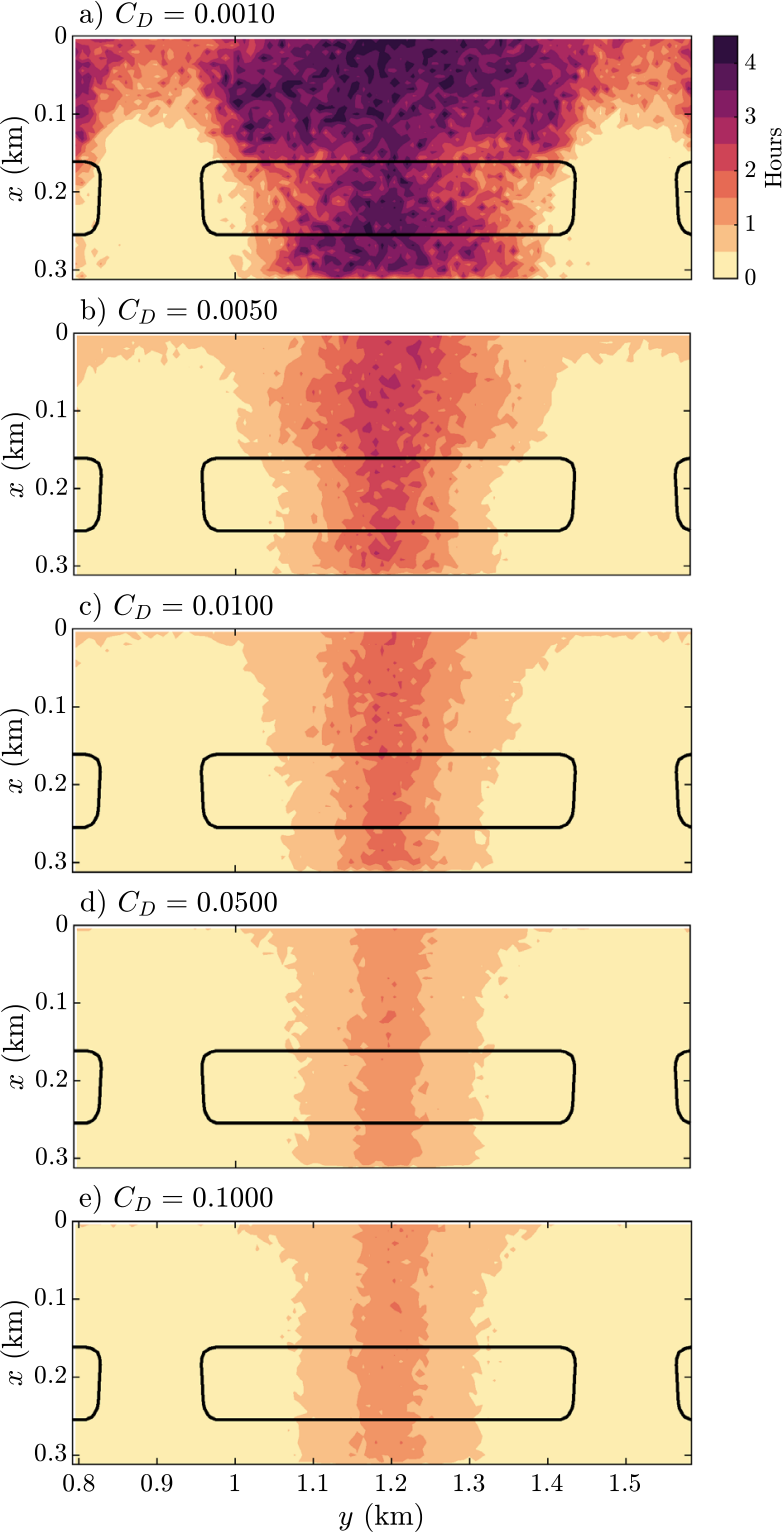
Depth-averaged residence times of particles released an hour before high tide. Solid black lines indicate reef flat location.

Figure 7 shows the residence times when releasing the particles an hour before high tide. The high reef flat roughness models (Fig. 7d,e) are not significantly affected, and most particles leave the system within 1 to 2 h. In contrast, in Fig. 7a,b the low reef flat roughness models show a significant increase in residence time sometimes exceeding five hours. Comparing to the cross-reef velocity profiles in Fig. 4, the models with high residence times are those with a pronounced undertow and horizontal circulation cells shoreward of the reef flat (Fig. 3b)

### Momentum phase diagram

**Figure 8:**
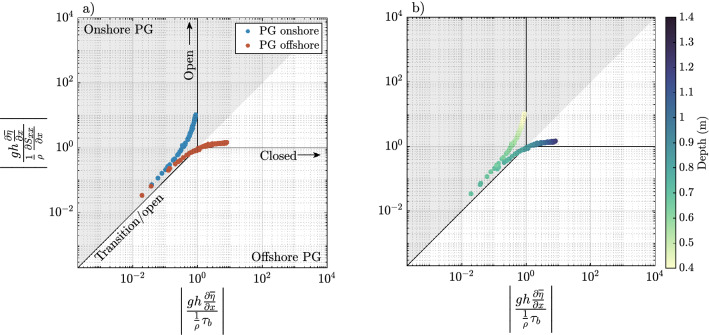
Momentum phase diagrams for the $C_D=0.01$ simulation. Momentum terms are evaluated over a tidal period at the location indicated in Fig. 2a on the reef flat. (**a**) Markers are colored according to the direction of the pressure gradient, i.e. an onshore (offshore) pressure gradient force is blue (red). (**b**) Markers are colored according to depth on the reef flat.

The three largest momentum terms on the reef flat were PGF, RSG, and BF. Advection and local acceleration were negligible. The relative importance of each term are presented in a momentum phase diagram^3^ in Fig. 8 for the $C_D=0.01$ model.

Data points along the vertical line $\left\| \frac{gh\frac{\partial {\overline{\eta }}}{\partial x}}{\frac{1}{\rho }\tau _b}\right\| =1$ indicate a balance between the pressure gradient force and bottom friction. This is typically associated with low tide conditions where the majority of incident wave energy is dissipated due to breaking on the forereef, and wave setup creates an onshore pressure gradient, i.e., an open reef. The cross-reef velocity, shown in Fig. 4c, is unidirectional and represented by an approximately logarithmic velocity profile, e.g., Eq. (2).

Data points along the horizontal line $\Vert \frac{gh\frac{\partial {\overline{\eta }}}{\partial x}}{\frac{1}{\rho }\frac{\partial S_{xx}}{\partial x}}\Vert =1$ indicate a balance between the pressure gradient force and the radiation stress gradient. This dynamic, referred to here as a closed reef, is indicative of beach-like dynamics and coincides with an offshore pressure gradient. This behavior occurs on high tide, where less wave energy is dissipated by depth-limited breaking.

**Figure 9:**
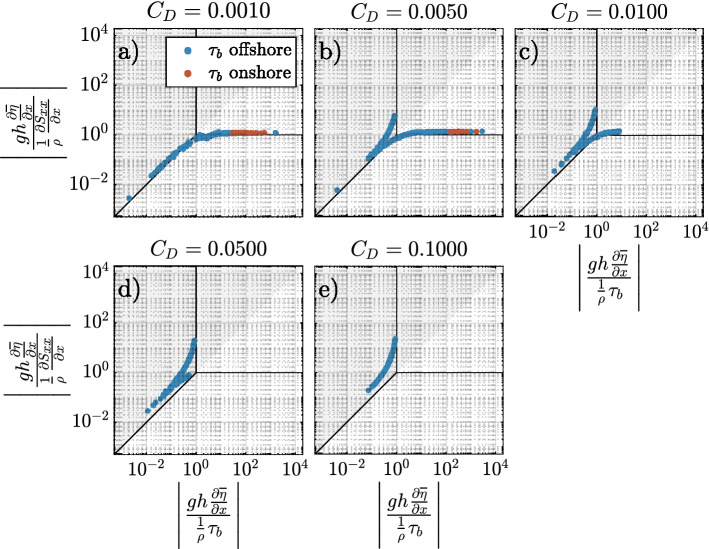
Momentum phase diagrams of simulations with varying reef flat drag coefficients. Momentum terms are evaluated over a tidal period at the location indicated in Fig. 2a. Markers are colored according to the direction of the bottom shear stress $\tau _b$.

All five models are summarized in Fig. 9. Here, the markers are colored according to the direction of the bottom stress. Hence, blue markers indicates the velocity along bottom is shoreward (offshore stress), and red markers indicate the bottom velocity is directed offshore (onshore stress). I.e., red markers are related to the presence of undertow and blue are related to the absence of undertow. Notably, when comparing between the models the undertow develops in the closed reef (beach-like) part of the phase diagram, and only in the models with low reef flat roughness.

### Sensitivity analysis

**Figure 10:**
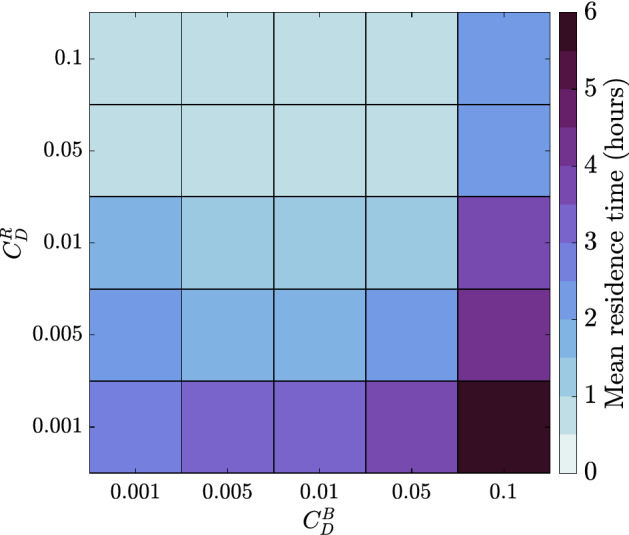
Mean residence time in hours on reef flat and in pools when initiated an hour before high tide. The superscripts *R* and *B* are used to differentiate between the drag coefficient on the reef flat, $C^R_{D}$, and everywhere else $C_D^B$ (‘background’).

In the previous results presented, the drag coefficient was varied on the reef flat only, and $C_D=0.01$ everywhere else. In this section, a sensitivity analysis is presented where both the drag coefficient on the reef flat ($C_D^R$ for ‘reef’, the area enclosed by the dashed lines in Fig. 2a) is varied as well as the drag coefficient in the rest of the domain ($C_D^B$ for ‘background’). All combinations of $C_D^{R},C_D^B\in [0.001,0.005,0.01,0.05,0.1]$ are run, resulting in a total of 25 models. Following the method in the “Residence times” section, residence times are calculated for each model, initiated an hour before high tide (i.e., $C_D^B=0.01$ and $C_D^R\in [0.001,0.005,0.01,0.05,0.1]$ would produce Fig. 7). Taking a mean of the residence time, the results are shown in Fig. 10.

Consistently, a higher reef flat roughness $C_D^R$ lowers residence times, in support of the main hypothesis presented in this paper. Generally, a higher background roughness $C_D^B$ tends to increase residence times, although this is not the case for the lowest value of $C_D^B$. This is an unexpected result which will be addressed in future work, however, it points to the non-trivial role of the pools and channels. In the case of low roughness on the reef flat and high background roughness, residence times increase significantly to approximately 6 h. The majority of the calculated residence times in this paper are less than 1–4 h, but given different geometry or forcing conditions this might exceed the dominant tidal period, and the effect of consecutive tidal cycles would become important as the system is not fully flushed over one 12 h period.

## Discussion

Results from idealized numerical models presented in this paper show how varying reef flat roughness on a reef can substantially change the circulation and thus the residence time on the reef. Models with high reef roughness and shallow reefs follow a classic reef circulation dynamic, with unidirectional, onshore reef flow that passes through deeper pools and channels alongshore behind the reef flat, and exits through channels transecting the reef flat. On the reef flat, an onshore pressure gradient is balanced by an offshore bed shear stress, and the flow is similar to open channel flow^3^. This dynamic, referred to as an open reef, is efficient at flushing the entire system (reef flat, pools, and channels) with residence times generally less than 2 h.

Models with low reef roughness and deeper reefs are characterized by sheared cross-reef flow, at times with a distinct offshore flow on the reef flat similar to beach undertow. On this reef flat, radiation stresses and barotropic pressure balance, referred to here as a closed (beach-like) reef. In spite of a strong offshore flow along the reef flat these models exhibit much longer residence times. This is in part due to strong vertical mixing offshore of the reef due to wave breaking which allows for recirculation, as well as entrapment in two counter-rotating horizontal eddies in the shoreward pools. Thus, the undertow is not effective at flushing the reef system and pools. Residence times for these models approached 5 h, not including the channels which consistently have a low residence time.

The fringing reef type studied here is representative of reefs across the globe and has been studied extensively previously (e.g.^2,7,12,20,21^). For these reefs, this study identifies a potential positive feedback mechanism where wide-spread bleaching events and coral death decreases reef roughness by lowering the geometric complexity of the sea bed, which in turn can transition a reef system from efficient flushing and short residence times to inefficient flushing and long residence times. Corals depend on their physical environment to bring in nutrients and cool offshore waters, and a less efficient circulation pattern could lead to further coral decline. Additionally, the tidal dependency found in this study shows that reefs that are currently open and efficiently flushing could transition to a closed, inefficiently flushing circulation pattern given sea level rise.

## Supplementary Information

Supplementary Figure 1.

Supplementary Figure 2.

Supplementary Video 1.

Supplementary Video 2.

Supplementary Legends.

## Data Availability

The model files developed for this study are available in the Stanford Digital Repository at https://purl.stanford.edu/fn366hf1765.
