# Supplementary figures and images for: Loss of reef roughness increases residence time on an idealized coral reef
Source: Sci Rep. 2022 Nov 12;12:19410. doi: 10.1038/s41598-022-24045-4 (PMC9653433; doi:10.1038/s41598-022-24045-4)

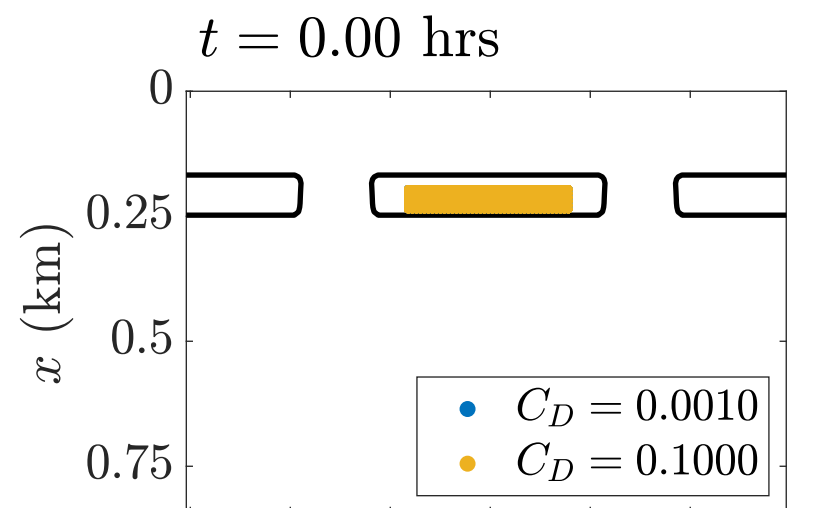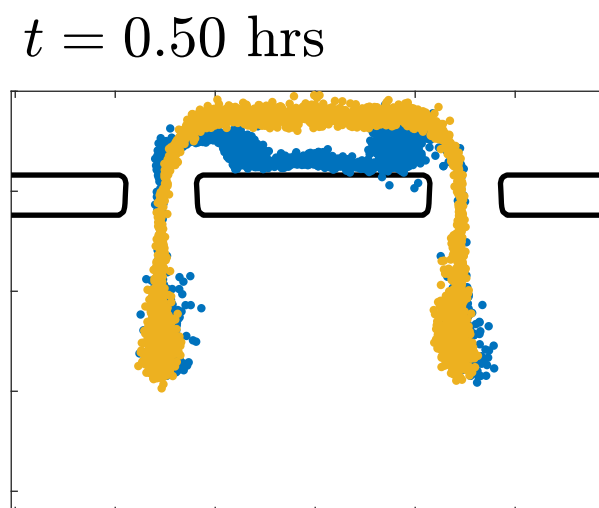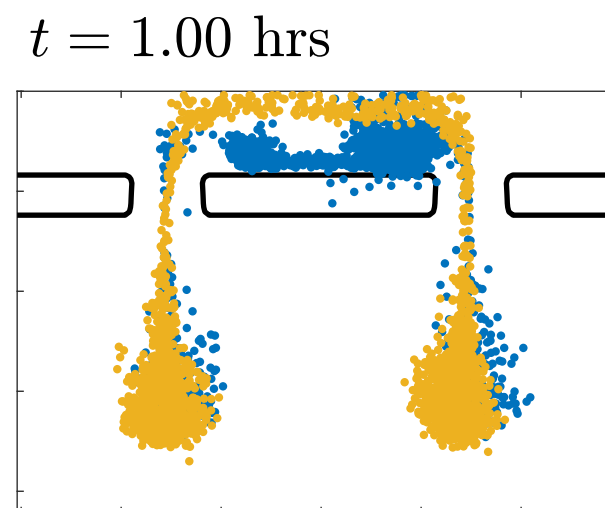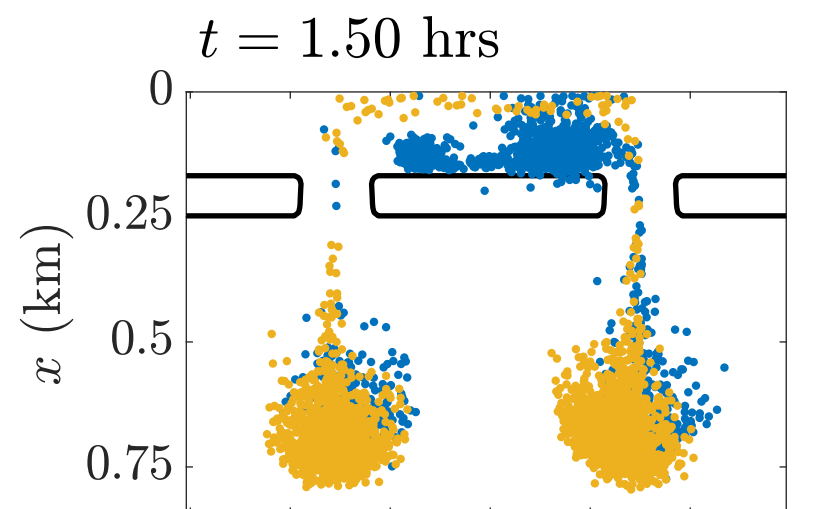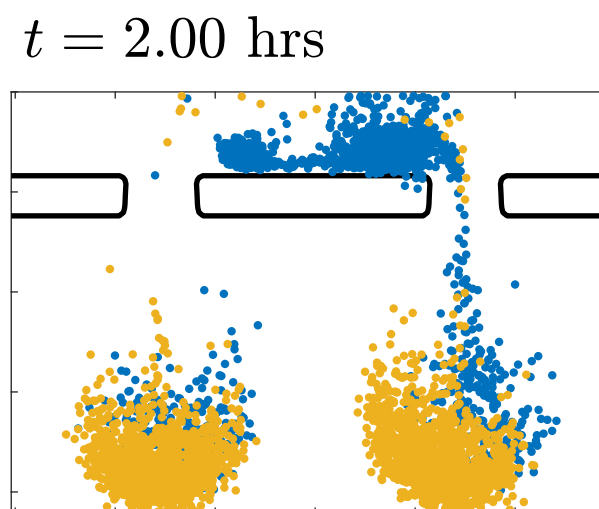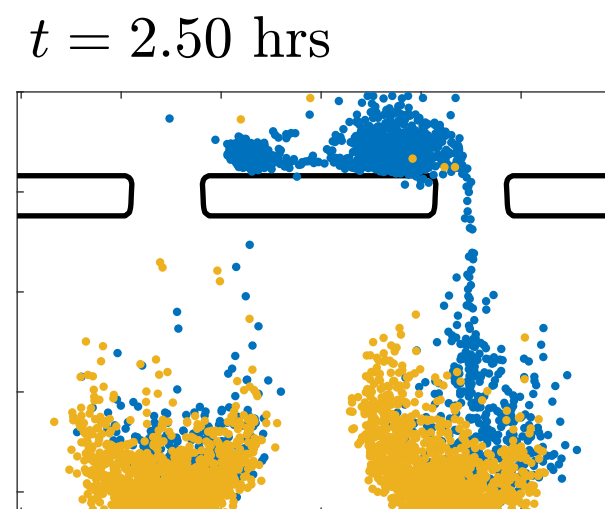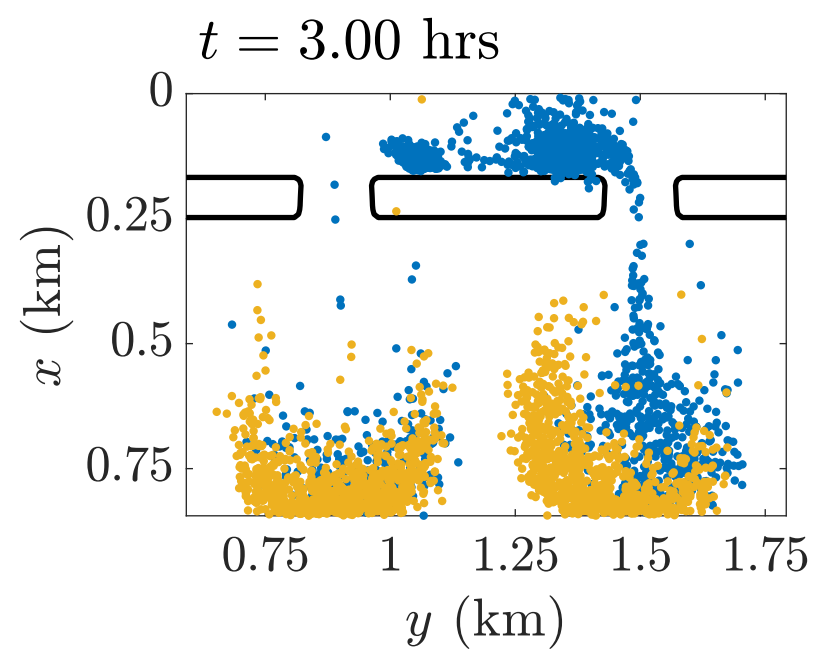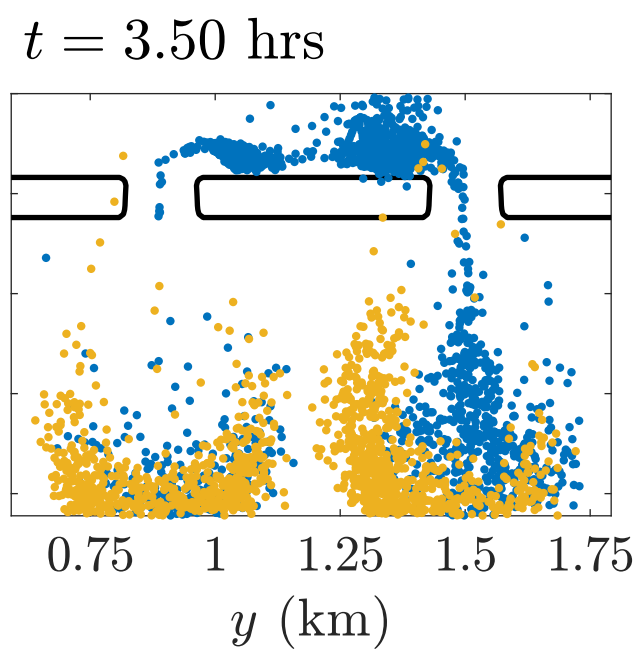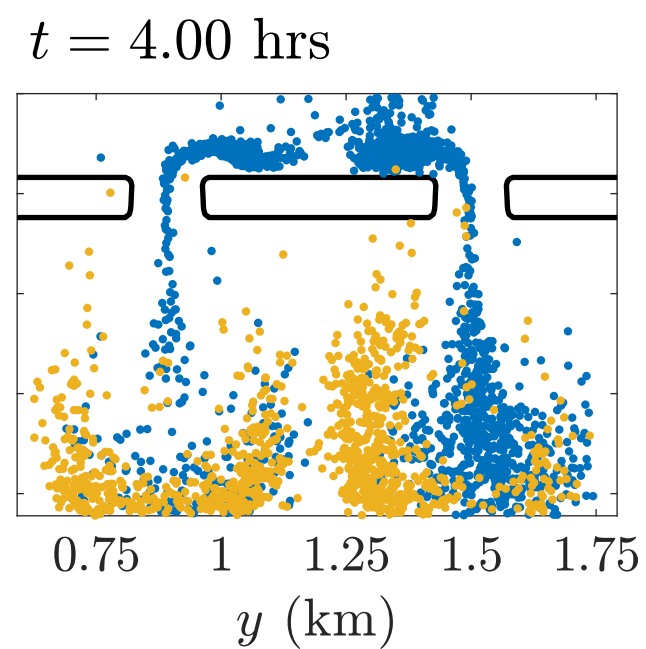

Supplement: Supplementary file 1 — Supplementary Figure 1. [file 41598_2022_24045_MOESM1_ESM.pdf]

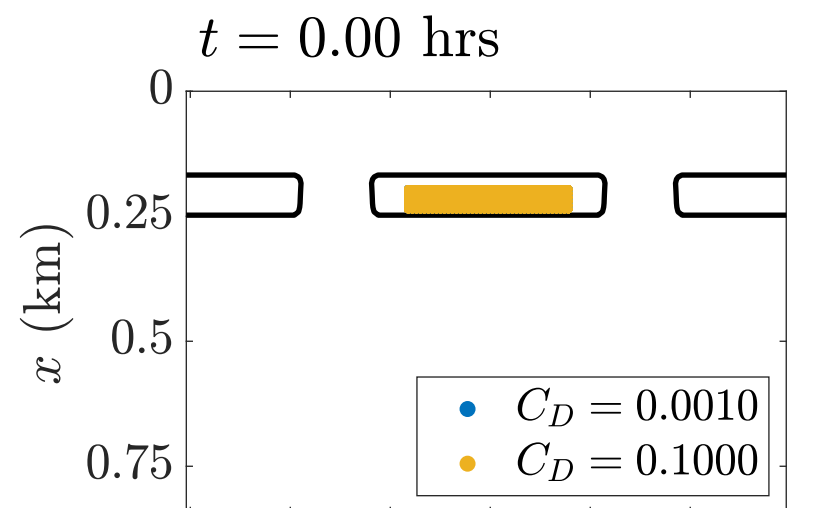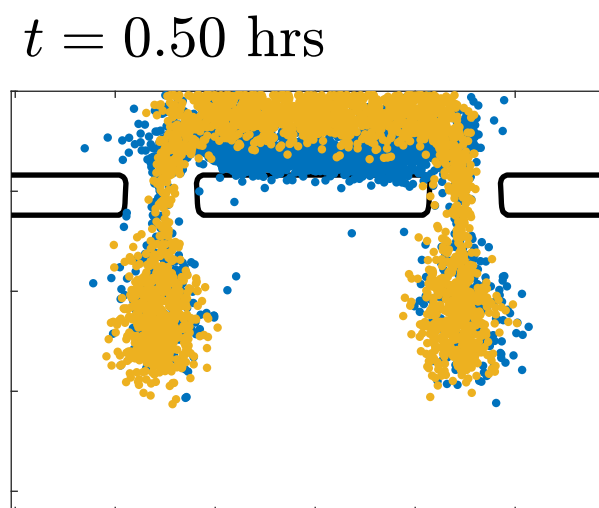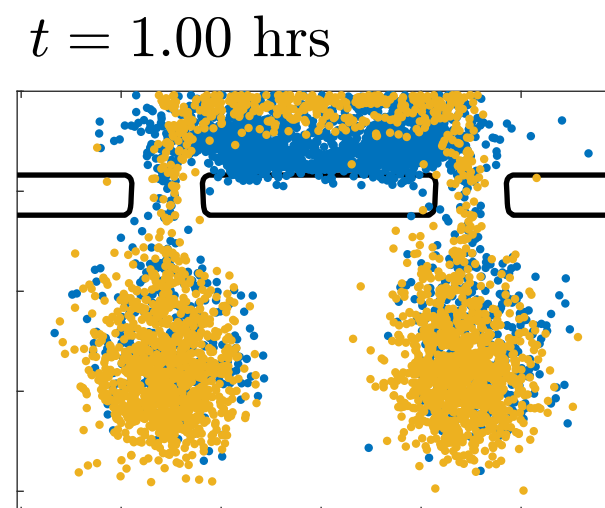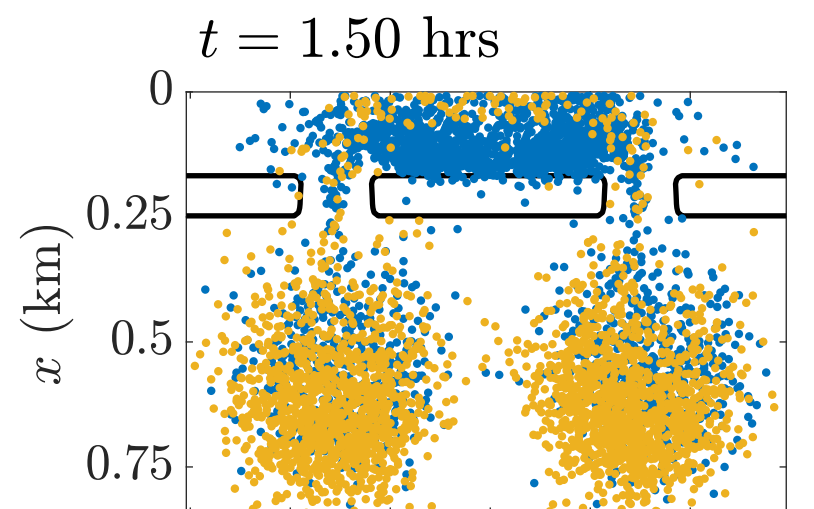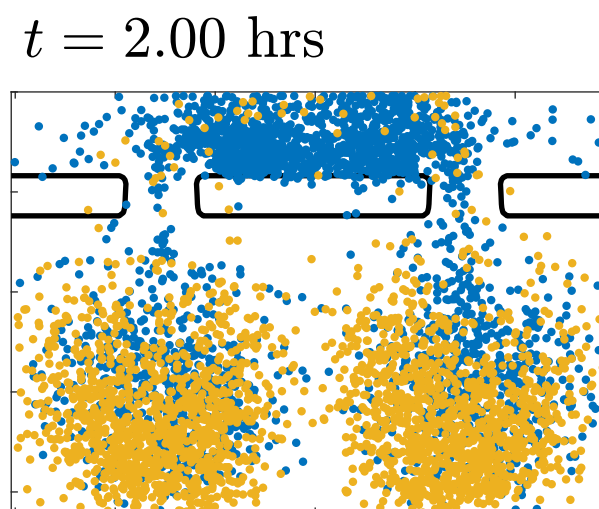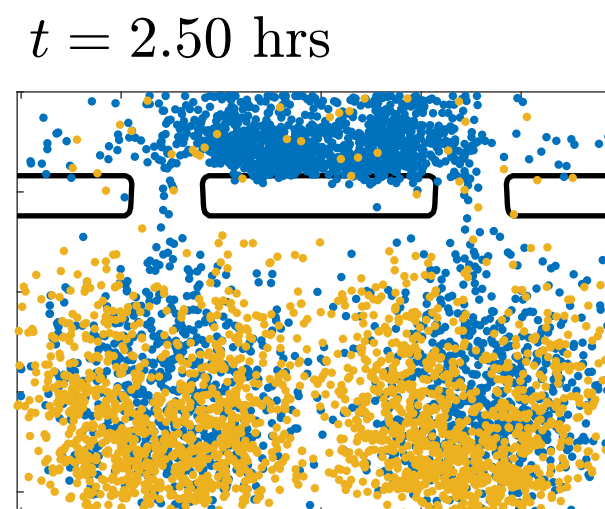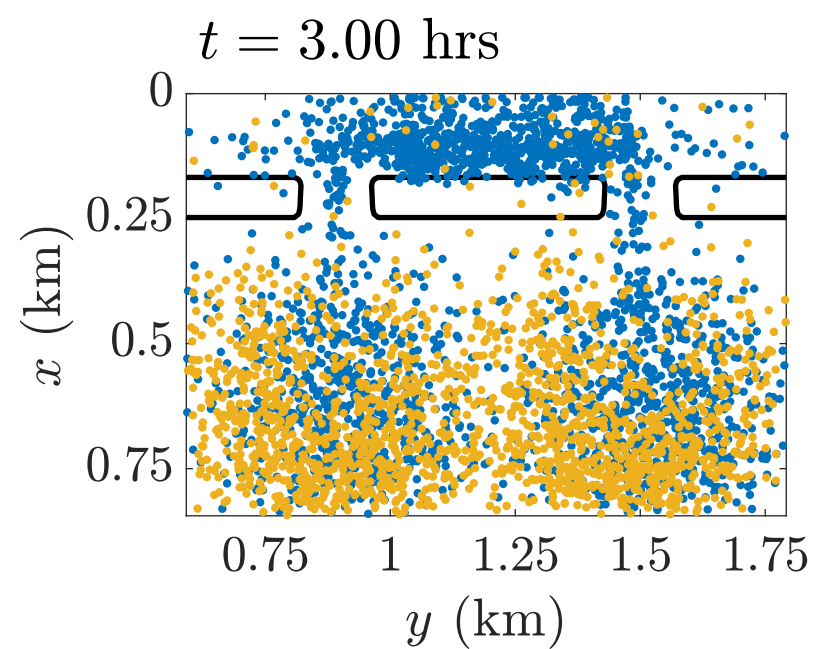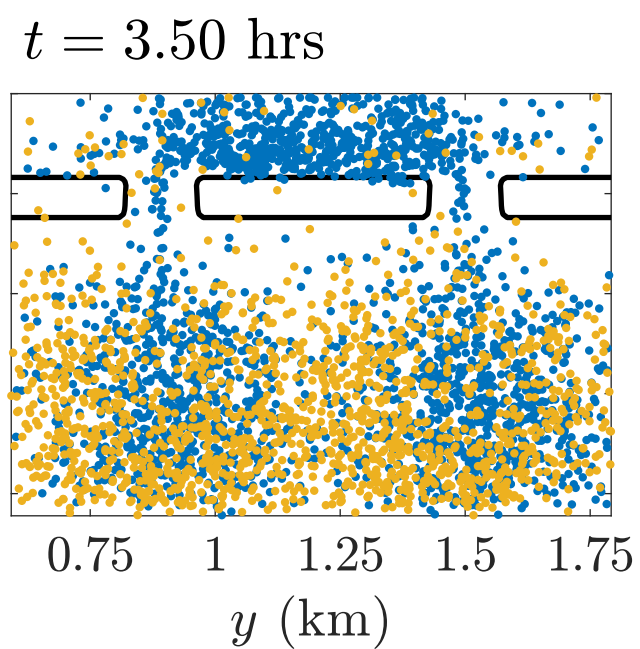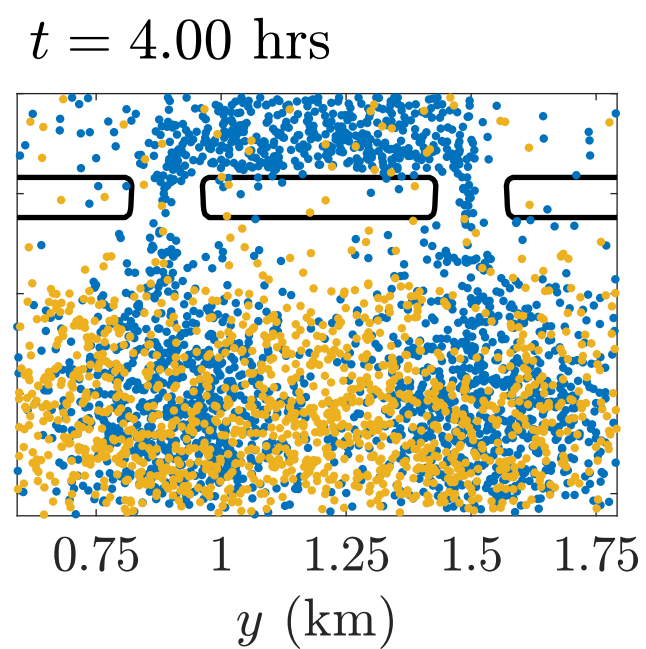

Supplement: Supplementary file 2 — Supplementary Figure 2. [file 41598_2022_24045_MOESM2_ESM.pdf]
