# Supplementary material for: Loss of reef roughness increases residence time on an idealized coral reef
Source: Sci Rep. 2022 Nov 12;12:19410. doi: 10.1038/s41598-022-24045-4 (PMC9653433; doi:10.1038/s41598-022-24045-4)
Supplement: Supplementary file 5 — Supplementary Legends. [file 41598_2022_24045_MOESM5_ESM.docx]

**Supplementary Information**

**Loss of reef roughness increases residence time on an idealized coral reef**

Lindhart, M.

**Supplementary Video 1**

Simulation of 20 particles released on the reef flat, in the low friction (Cd=0.001) model on high tide. The particles are located in two (x,y)-points, with one particle in the center of each vertical layer, darker particles are initiated at the top of the water column and lighter at the bottom. Trajectories are calculated according to equation 5 and section 2.3.

**Supplementary Video 2**

Simulation of 20 particles released on the reef flat, in the high friction (Cd=0.1) model on high tide. The particles are located in two (x,y)-points, with one particle in the center of each vertical layer, darker particles are initiated at the top of the water column and lighter at the bottom. Trajectories are calculated according to equation 5 and section 2.3.

**Supplementary Figure 1**

Figure 5 replicated with a smaller horizontal eddy viscosity of Kh = 0.1m^2/s.

**Supplementary Figure 2**

Figure 5 replicated with a larger horizontal eddy viscosity of Kh = 1m^2/s.
